# Association Between Dieting Failure and Unconscious Hedonic Responses to Food

**DOI:** 10.3389/fpsyg.2020.02089

**Published:** 2020-09-04

**Authors:** Wataru Sato

**Affiliations:** Psychological Process Team, BZP, RIKEN, Kyoto, Japan

**Keywords:** dieting, food, subliminal affective priming, perceived self-regulatory success in dieting scale, unconscious emotion

## Abstract

Dieting is a popular but difficult strategy for reducing weight. Previous studies have revealed several psychological characteristics associated with dieting failure. Here, the hypothesis that dieting failure is associated with unconscious hedonic responses to food was tested with a subliminal affective priming task. Food image primes or their scrambled mosaic primes were subliminally presented. Participants scored their liking of the subsequent target ideographs. The participants’ subjective dieting success was additionally assessed using questionnaires. Differences in liking scores of target ideographs between the food and mosaic conditions, as well as liking scores of target ideographs under the food condition by partialing-out other effects, were negatively associated with dieting success scores. These results suggest that dieting failure is associated with the strength of unconscious hedonic responses to food and that environmental controls to reduce food cues may be needed for dieting success.

## Introduction

Dieting is a popular but difficult strategy for reducing weight. Survey studies have shown that dieting is common during the entire lifespan in both females and males (Slof-Op’t Landt et al., 2017). However, in contrast to dieters’ expectation of weight reduction, empirical studies consistently revealed that dieting often fails and may lead to weight gain, which could induce health problems (for a review, see Lowe et al., 2013). The data suggest that dieting is associated with weight dissatisfaction, rather than with actual weight; hence, even underweight individuals may try dieting (Eik-Nes et al., 2015). Dieting was also shown to be a risk factor for eating disorders (e.g., Stice et al., 2017; for a review, see Lowe and Timko, 2004). These data suggest that, to promote physical and mental health, further research is warranted to understand and improve dieting practices.

Several studies have explored psychological mechanisms underlying dieting (for a review, see Elfhag and Rössner, 2005). These studies identified various psychological characteristics that were associated with dieting failure, including high trait impulsiveness (Meule et al., 2012), high trait food craving (Meule et al., 2011), low inhibitory control (Houben et al., 2012), low goal-based self-regulation (Fishbach et al., 2003), and personal history of dieting (Lowe et al., 2006, 2013, 2019). However, because dieting is a complex phenomenon related to multiple psychological, environmental, and biological factors (Maclean et al., 2011), it is highly plausible that additional psychological processes contribute to dieting failure.

One psychological process potentially related to dieting that remains untested is unconscious hedonic responses to food. One previous study has demonstrated that food stimuli can elicit unconscious hedonic responses (Sato et al., 2016). That study used a subliminal affective priming task (Murphy and Zajonc, 1993) and demonstrated that food images presented subliminally heightened the liking scores of subsequently presented targets (i.e., neutral faces) more than the subliminally presented mosaic images did, suggesting that the hedonic reactions triggered by food images unconsciously spilled over into the appraisal of unrelated targets. Their results further showed that the preference for subliminally presented food images was negatively correlated with scales measuring restrained eating on the Dutch Eating Behavior Questionnaire (van Strien et al., 1986), which assesses intentions to restrict food intake and actual behavioral restraint (Larsen et al., 2007). However, interpretation of this result is difficult because that scale was originally developed to assess a tendency toward paradoxical overeating (van Strien et al., 1986) and does not explicitly assess motivations or behaviors aimed at weight loss (Lowe et al., 2013). Furthermore, empirical findings regarding the relationship between such scales and dieting performance remain inconsistent (for a review, see Lowe et al., 2013). One may expect that scales like the perceived self-regulatory success in dieting scale (PSRS; Fishbach et al., 2003), which directly assesses dieting success or failure and was validated by consistent medium-to-high negative correlations with body mass index (BMI) across studies (Meule et al., 2012) may provide more clear-cut evidence. Based on these data, together with abundant evidence that hedonic responses to food generally motivate food consumption (for a review, see Søensen et al., 2003), it was hypothesized that individuals’ tendency toward dieting failure could be associated with the strength of their unconscious hedonic responses to food.

**Figure 1:**
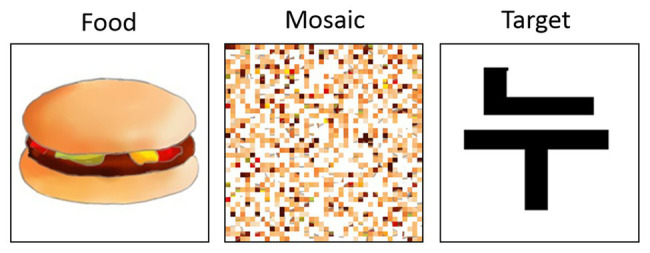
Illustrations of food and mosaic images and target ideographs. In the actual experiment, food, and mosaic images were photographs. Participants scored their liking of target ideographs, which were primed by subliminally presented food or mosaic images under the subliminal condition. They scored their liking of food or mosaic images under the supraliminal condition.

This hypothesis was tested by assessing participants’ unconscious hedonic responses to food using a subliminal affective priming task, as in a prior study (Sato et al., 2016). Food image primes or their scrambled mosaic primes were presented for 30 ms in the peripheral visual field (i.e., outside participants’ attentional focus), and nonsense ideograph targets were then presented (Figure 1). The participants scored their liking of the targets, which could be modulated by the primes. The participants’ conscious hedonic responses were also assessed by measuring their liking of supraliminally presented food and mosaic images. In addition, participants’ subjective evaluations of dieting success were assessed using the PSRS (Fishbach et al., 2003). The association between hedonic responses to food under each presentation condition and PSRS scores was evaluated.

## Materials and Methods

### Participants

Participants were 29 healthy Japanese volunteers (15 females; mean ± SD age, 21.6 ± 2.0 years). The required sample size was determined based on an *a priori* power analysis implemented with G*Power 3.1.9.2 (Faul et al., 2009). Correlation coefficients (one-tailed) at a strong effect size (*ρ* = 0.5) with *α* of 0.05 and power (1 − *β*) of 0.80 required more than 21 participants. The participants were recruited through advertisements on notice boards at Kyoto University. None of the participants had any deficiency in visual acuity were familiar with Korean characters (i.e., the targets), and knew the research objective. All participants were of normal weight (i.e., <25 BMI; actual mean ± SD BMI, 21.3 ± 1.8 kg/m^2^). The participants were instructed to fast for >3 h before the experiment. Participants’ hunger level, measured using a five-point scale ranging from 1 (not at all) to 5 (very), was moderate (mean ± SD, 2.3 ± 0.6). All participants provided written informed consent to participate in this study. This study received ethical approval from the Ethics Committee of the Unit for Advanced Studies of the Human Mind, Kyoto University.

### Experimental Design

The experiment was constructed as a one-factor within-participants design, with a stimulus-type factor having two levels (food and mosaic) under each of the subliminal and supraliminal presentation conditions. Dependent variables were (1) the liking scores of target ideographs, which were primed by subliminally presented food or mosaic images, under the subliminal condition; (2) the liking scores of food or mosaic images under the supraliminal conditions; and (3) PSRS scores.

### Stimuli

Food images were 24 color photographs of food, including three images of each of eight food types (hamburgers, fried chicken, pizza, doughnuts, sushi, roast fish, mixed rice, and noodles), selected from images presented on websites (Figure 1). The images were cropped using PhotoShop CS6 (Adobe, San Jose, CA, United States). Mosaic images were constructed from the food images using MATLAB 6.5 (MathWorks, Natick, MA, United States). All food images were divided into 1,600 small squares, which were then randomly reordered. A mosaic pattern comprising fragments of food images not used in the mosaic condition was also created as a mask. Target ideographs under the subliminal condition were 48 images of Korean characters, which were shown to be emotionally neutral (Sato and Aoki, 2006). The size of all stimuli was 5.0° × 5.0°of visual angle.

### Apparatus

Experiments were controlled by presentation (Neurobehavioral Systems, Berkeley, CA, United States) implemented on a Windows computer (HP Z200 SFF; Hewlett-Packard Japan, Tokyo, Japan). The stimuli were presented on a 19-inch cathode ray tube display (HM903D-A; Iiyama, Tokyo, Japan) at a refresh rate of 100 Hz and a resolution of 1024 × 768 pixels.

### Questionnaire

The PSRS (Fishbach et al., 2003) was used to measure the participants’ subjective evaluations of dieting success. Using three items and seven-point scales, participants rated their success at watching their weight and losing weight and their difficulty with staying in shape. Items were as follows: “How successful are you in watching your weight?”; “How successful are you in losing extra weight?”; and “How difficult do you find it to stay in shape?” A previous study used the PSRS and demonstrated good reliability and validity (Meule et al., 2012). The English-language version was translated into Japanese by a translator, and translation was validated through back-translation by an independent translator.

### Procedure

The experiments were run individually in a soundproof room (Science Cabin, Takahashi Kensetsu, Tokyo, Japan). Participants were instructed that the experiment concerned image appraisals. The participants first filled out a set of questionnaires, including a measure of their hunger level and the PSRS. They were then seated 0.57 m from the display and performed liking scoring under the subliminal and supraliminal conditions.

Ninety-six trials involving liking scoring (24 food images and 24 mosaics for left and right visual fields) were conducted under the subliminal condition and 96 under the supraliminal condition. A short break was interposed after 48 trials. The order of presentation of conditions was fixed, with the subliminal condition first and the supraliminal condition second, because the primary objective of this study was to investigate unconscious hedonic responses to food under the subliminal condition without conscious recognition of food processing. The order of stimulus conditions (food and mosaic) was randomized within each presentation condition. Five practice trials were given for each presentation condition.

In each trial under the subliminal condition, after a fixation point was presented for 1,000 ms at the center of the visual field, a prime (a food or mosaic image) was presented for 30 ms in either the left or right visual field (the inside edge was 5° peripheral to the center). This was followed by a mask presented at the identical location for 170 ms. Immediately after that, a target ideograph was presented in the same place for 1,000 ms. Finally, a scoring panel was displayed until the participant finished responding. Participants were asked to gaze at the fixation point and to score their liking of the target ideograph using a nine-point scale from 1 (not at all) to 9 (very much) by pressing typing (alphanumeric) keys with their right forefinger.

In each trial under the supraliminal condition, after a fixation point was presented for 1,000 ms at the center of the visual field, a food or mosaic image was shown for 200 ms in either the left or right visual field (the inside edge was 5° peripheral to the center). After a blank period of 1,000 ms, the scoring panel appeared until the participant finished responding. The participants were instructed to gaze at the fixation point and to score their liking of the food/mosaic image in the same manner as in the subliminal condition.

After the liking scoring tasks, participants performed the forced-choice discrimination task to objectively assess the conscious awareness of stimuli under the subliminal condition. A total of 48 trials were conducted using only the food images. In each trial, an image was presented as in the subliminal condition, and then two food images (one of which had already been presented) were presented. The participants were asked to select the presented image. A one-sample *t*-test (two-tailed) confirmed that the correct discrimination rate was not significantly different from chance [mean ± SE% correct, 54.0 ± 3.6; *t*(28) = 1.12, *p* > 0.1]. Subsequent interviews further showed that none of the participants consciously perceived the food images under the subliminal condition. After debriefing, all the participants gave permission for the analysis of their data.

### Data Analysis

Data were analyzed using SPSS 16.0J (SPSS Japan, Tokyo, Japan). The liking scores under each presentation condition were analyzed separately. As an index of food liking, controlling for general response patterns (e.g., high scores for all items), liking difference scores (c.f. Bedeian et al., 1994) between the food and mosaic conditions were calculated. The liking differences were first analyzed using one-sample *t*-tests (one-tailed). Then, correlation coefficients between the liking difference scores and the PSRS scores were calculated and tested for their significance (one-tailed). To test the effects of age, sex, BMI, and hunger level, partial correlations controlling for these measures were also calculated, as well as the correlation coefficient between these measures and liking difference scores or PSRS scores. Although the hypothesis addressed only results under the subliminal condition, the data for the supraliminal condition were analyzed in the same manner for descriptive purposes. In addition, because debate regarding the reliability and validity of difference scores persists (Bedeian et al., 1994; Griffin et al., 1999), polynominal regression analyses (Edwards and Parry, 1993) were conducted with PSRS scores as the dependent variable and liking scores for food images and mosaics and their squared and product terms as the independent variables. The β values of the liking scores for food images were evaluated for differences from zero (one-tailed); other β values were also tested exploratorily. A value of *p* < 0.05 was considered significant. As preliminary data analysis, the Mahalanobis distance for each analysis was calculated to identify bivariate outliers. No significant outlier was identified for any analysis of difference scores and partialed-out liking scores under the subliminal and supraliminal conditions (*d* < 3.5, *p* > 0.10).

## Results

**Figure 2:**
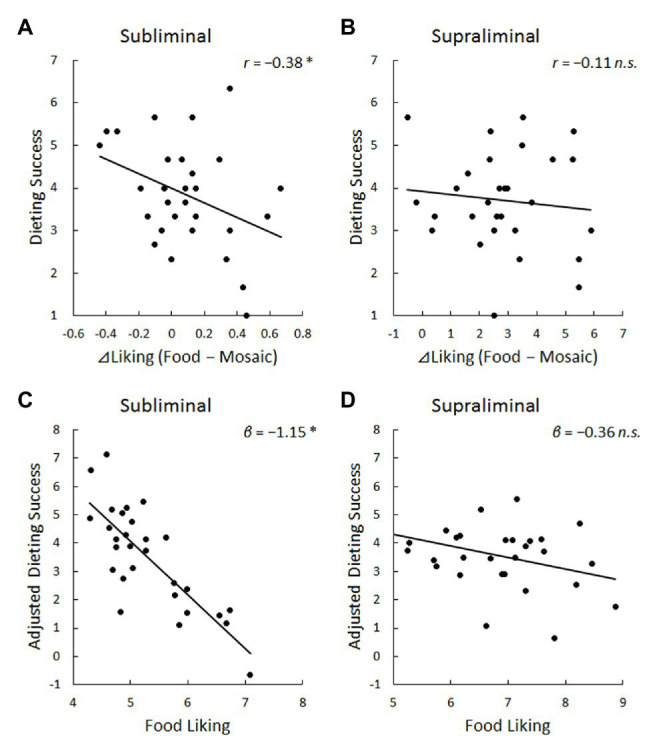
Scatter plots and regression lines for the relationship of dieting success scores (or their adjusted values) with liking difference scores (i.e., food–mosaic) under the subliminal **(A)** and supraliminal **(B)** presentation conditions and food liking scores under the subliminal **(C)** and supraliminal **(D)** presentation conditions. Dieting success was assessed using the perceived self-regulatory success in dieting scale (PSRS). Adjusted values were derived by partialing-out the effects of no interest (i.e., liking scores for mosaics and squared and product terms for liking scores for food and mosaics). Liking scores under the food condition (food liking) were assessed indirectly (for target ideographs after food primes) under the subliminal condition and directly (for food images) under the supraliminal condition. ^*^ *p* < 0.05; *n.s.* = not significant.

Under the subliminal condition, the mean ± SE liking scores of target ideographs under the food and mosaic conditions were 5.31 ± 0.14 and 5.22 ± 0.13, respectively. The liking difference scores of target ideographs between the food and mosaic conditions were significantly different from zero [mean ± SE, 0.09 ± 0.05, *t*(28) = 1.73, *p* < 0.05]. A negative and significant correlation was found between the liking difference scores and PSRS scores (*r* = −0.38, *p* < 0.05; Figure 2, upper left). Partial correlation analyses showed that controlling for age, sex, BMI, and hunger level did not change the significant correlation between the liking difference scores and PSRS scores (*pr* < −0.32, *p* < 0.05), and none of these variables were significantly correlated with either liking difference or PSRS scores (|*r*| < 0.24, *p* > 0.10). A polynomial regression analysis with PSRS scores as the dependent variable and liking scores of target ideographs under the food and mosaic conditions and their squared and product terms as the independent variables revealed that only the β value of liking scores of target ideographs under the food condition was significant [*β* = −1.15, *t*(23) = 2.03, *p* < 0.05; Figure 2, lower left].

Under the supraliminal condition, the mean ± SE liking scores of food and mosaic images were 6.84 ± 0.18 and 4.01 ± 0.25, respectively. The liking difference scores were significantly different from zero [mean ± SE, 2.83 ± 0.31, *t*(28) = 9.02, *p* < 0.001]. The correlation coefficient between the liking difference and PSRS scores was not significant (ns; *r* = −0.11, *p* > 0.10; Figure 2, upper right). Partial correlation coefficients controlling for age, sex, BMI, and hunger level were also not significant (|*pr*| < 0.08, *p* > 0.10), and none of these were significantly correlated with either the liking difference or PSRS score (|*r*| < 0.22, *p* > 0.10). A polynomial regression analysis showed no significant association between PSRS scores and liking scores of food images [*β* = −0.36, *t*(23) = 1.57, *p* > 0.10; Figure 2, lower right].

## Discussion

The results revealed that the liking scores of target ideographs under the subliminal condition differed between the food and mosaic prime conditions on average, although the difference was small and varied substantially across individuals. The results are consistent with those of previous studies (e.g., Sato et al., 2016) and support the presence of unconscious hedonic responses to food.

More important, the results under the subliminal condition demonstrated that the liking difference scores of target ideographs between the food and mosaic conditions, as well as the liking scores of target ideographs under the food condition partialing-out other effects were negatively associated with the dieting success scores. This result is in line with the finding of a previous study (Sato et al., 2016) that differences in liking scores of targets after subliminally presented food vs. mosaic images were negatively correlated with scores on the restraint eating scale (van Strien et al., 1986). However, debates about whether such a scale reflects actual dieting persist (Lowe et al., 2013). No previous study has investigated the association between dieting success *per se* and unconscious hedonic responses to food. This study newly demonstrated that the strength of unconscious hedonic responses to food is associated with dieting failure.

The results under the supraliminal condition showed no significant association between conscious hedonic responses to food and dieting failure. This result is consistent with the previous finding that liking differences between supraliminally presented food and mosaic images did not show evident associations with eating tendencies (Sato et al., 2016). This result suggests that conscious hedonic appraisals of food differ from unconscious processing of food by introducing the role of cognitive appraisal, which is not closely associated with dieting performance. The results can be interpreted in general perspectives that unconscious and conscious processing can be dissociable (Kahneman, 2011) and that certain elements of daily eating behaviors are controlled unconsciously (Wansink, 2010).

Our results have practical implications. The results show that individuals who are prone to failing at dieting have a strong tendency toward unconscious hedonic responses to food. Related to this result, previous psychological findings have revealed that unconscious emotional responses are only minimally influenced by conscious reflection (Murphy and Zajonc, 1993). Neuroscientific data showed that unconscious processing cannot be inhibited by the conscious will (Filevich et al., 2013). Together with these data, the present result suggests that individuals who are prone to failing at dieting should anticipate and prepare for the irresistible, unconscious hedonic responses to food that foster food consumption and weight gain. Environmental controls that reduce food cues, a strategy frequently used in cognitive behavioral therapies for eating control (Costain and Croker, 2005), may be recommended.

Plausible neural substrates for the association between dieting failure and unconscious hedonic processing of food involve the amygdala. A previous functional neuroimaging study has found that the amygdala was activated in response to subliminally presented food images *via* the subcortical visual pathway (Sato et al., 2019). Another neuroimaging study reported that the combined activity of five brain regions, including the amygdala, predicted BMI (Killgore et al., 2013). In future research, it may be helpful to investigate task-related or resting-state amygdala activity associated with dieting failure so that we can acquire biomarkers to objectively assess and intervene in dieting.

Several limitations of this study should be addressed. First, only subjective ratings of dieting success, which could be biased, were used. Future investigations with objective measures of dieting performance, such as weight records, are required to confirm rigorously the association between dieting performance and unconscious hedonic responses to food.

Second, our sample size was small and therefore may have lacked the power to detect some effects, such as the relationship between dieting success scores and BMI, which was found in a previous survey with a larger sample (*n* > 300; Meule et al., 2014), and the association between dieting success scores and supraliminal liking scores for food. Additionally, the small sample size made it difficult to remove outliers effectively, despite their influence (Cousineau and Chartier, 2010). Further studies with larger samples would be helpful to investigate these phenomena.

Third, the presentation condition order was fixed, which may have affected findings under the supraliminal condition due to habituation. Although the fixed order was used to prevent conscious food processing under the subliminal condition, counterbalanced presentation orders would be needed to contrast unconscious with conscious hedonic food processing rigorously.

Finally, because the experimental design was correlational, no causal relationship between unconscious hedonic response to food and dieting success can be inferred. Interestingly, a previous study has reported that intervention in unconscious hedonic responses to food through subliminal evaluative conditioning (i.e., association between subliminally presented non-food negative images and food images) resulted in lower liking ratings for food, which persisted for several days (Legget et al., 2015). Together with these data, the present results suggest the possibility that intervention in unconscious food processing may change dieting performance, which could contribute to our understanding of the causal mechanisms underlying dieting and thereby improve the well-being of many people.

## Data Availability Statement

All datasets presented in this study are included in the article/Supplementary Material.

## Ethics Statement

The studies involving human participants were reviewed and approved by Ethics Committee of the Unit for Advanced Studies of the Human Mind, Kyoto University. The patients/participants provided their written informed consent to participate in this study.

## Author Contributions

WS designed the research, obtained and analyzed the data, and wrote the manuscript.

### Conflict of Interest

The author declares that the research was conducted in the absence of any commercial or financial relationships that could be construed as a potential conflict of interest.
